# Phenotypic Classification of Eye Colour and Developmental Validation of the Irisplex System on Population Living in Malakand Division, Pakistan

**DOI:** 10.3390/biomedicines11041228

**Published:** 2023-04-20

**Authors:** Murad Ali Rahat, Fazal Akbar, Akhtar Rasool, Muhammad Ilyas, Allah Rakha, Sulaiman Shams, Musharraf Jelani, Fehmida Bibi, Bader H. Shirah, Angham Abdulrhman Abdulkareem, Muhammad Imran Naseer, Muhammad Israr

**Affiliations:** 1 Centre for Biotechnology and Microbiology, University of Swat, Charbagh 19120, Pakistan; 2 Department of Forensic Sciences, University of Swat, Charbagh 19120, Pakistan; 3 Centre for Omic Sciences, Islamia College University Peshawar, Peshawar 25120, Pakistan; 4 Department of Forensic Sciences, University of Health Sciences, Lahore 54600, Pakistan; 5 Department of Biochemistry, Abdul Wali Khan University Mardan, Mardan 23200, Pakistan; 6 Special Infectious Agents Unit, King Fahd Medical Research Centre, King Abdulaziz University, Jeddah 21589, Saudi Arabia; 7 Department of Medical Laboratory Technology, Faculty of Applied Medical Sciences, King Abdulaziz University, Jeddah 21589, Saudi Arabia; 8 Department of Neuroscience, King Faisal Specialist Hospital & Research Centre, Jeddah 21589, Saudi Arabia; 9 Faculty of Science, Department of Biochemistry, King Abdulaziz University, Jeddah 21589, Saudi Arabia; 10 Center of Excellence in Genomic Medicine Research, King Abdulaziz University, Jeddah 21589, Saudi Arabia

**Keywords:** eye colour, Irisplex, genotypes, Pakhtoon population, Malakand Division

## Abstract

The core objective of forensic DNA typing is developing DNA profiles from biological evidence for personal identification. The present study was designed to check the validation of the IrisPlex system and the Prevalence of eye colour in the Pakhtoon population residing within the Malakand Division. Methods: Eye colour digital photographs and buccal swab samples of 893 individuals of different age groups were collected. Multiplexed SNaPshot single base extension chemistry was used, and the genotypic results were analysed. Snapshot data were used for eye colour prediction through the IrisPlex and FROG-kb tool. Results: The results of the present study found brown eye colour to be the most prevalent eye colour in comparison to intermediate and blue coloured. Overall, individuals with brown-coloured eyes possess CT (46.84%) and TT (53.16%) genotypes. Blue eye-coloured individuals are solely of the CC genotype, while individuals of intermediate eye colour carry CT (45.15%) and CC (53.85%) genotypes in rs12913832 SNP in the *HERC2* gene. It was also revealed that brown-coloured eyes individuals were dominant among all age groups followed by intermediate and blue. Statistical analysis between particular variables and eye colour showed a significant *p*-value (<0.05) for rs16891982 SNP in *SLC45A2* gene, rs12913832 SNP in *HERC2* gene, rs1393350 SNP in *SLC45A2*, districts and gender. The rest of the SNPs were non-significant with eye colour, respectively. The rs12896399 SNP and SNP rs1800407 were found significant with rs16891982 SNP. The result also demonstrated that the study group differs from the world population based on eye colour. The two eye colour prediction results were compared, and it was discovered that IrisPlex and FROG-Kb had similar higher prediction ratios for Brown and Blue eye colour. Conclusions: The results of the current study revealed brown eye colour to be the most prevalent amongst members of the local population of Pakhtoon ethnicity in the Malakand Division of northern Pakistan. A set of contemporary human DNA samples with known phenotypes are used in this research to evaluate the custom panel’s prediction accuracy. With the aid of this forensic test, DNA typing can be supplemented with details about the appearance of the person from whom the sample was taken in cases involving missing persons, ancient human remains, and trace samples. This study may be helpful for future population genetics and forensics studies.

## 1. Introduction

Iris colour as a multifactorial hereditary trait varies depending on the race and ethnicity of people. Pigmentation of the eye is due to a polymer known as melanin, consisting of Eumelanin and Pheomelanin controlling normal pigmentation. Eumelanin is present in a black-brownish colour that is present in dark hairs, eyes and skin. Opposite to the Eumelanin, the Pheomelanin is yellow reddish in colour and found in light hairs, eyes and light skin colour [1,2,3]. For eye pigmentation, the ratio of eumelanin and pheomelanin plays a key role. Eumelanin is the main pigment in most eye colours while pheomelanin is present only in traces. For example, the concentration of pheomelanin is the same in both blue and brown eyes [4]. The exception is made to this in green eyes where a concentration of pheomelanin is higher as compared to eumelanin [5]. Other than colour, other differentiating biochemical properties are present in eumelanin and pheomelanin. Eumelanin is photo protective and an antioxidant protecting against harmful UV rays. In contrast, pheomelanin is photo reactive and oxidative [6]. To date, multiple studies have been conducted to develop a universal classification for eye colour. Initially, eye colours were classified into blue, grey, black and auburn categories by Pétrequin in 1843. Later on in 1854, the categories of blue eye colour were expanded into gray, green and blue, while brown eye colour categories are black shades, dark brown, hazel and auburn [7]. A colour chart was developed in 1903 and consisted of 16 eye colours from dark brown (number 1) to light blue (number 16) [8]. Later on, standardized computerizes colours were used against digital images [9]. In 2008, a study proposed a 24-scale classification from 1 (least pigmentation) to 24 (most pigmentation), which is like to the previous Martin’s eyes [10,11]. Despite all of these, a new stem of eye colour classification is used and classified eye colour into blue, brown and intermediate based on eye colour photographed [12]. Eye colour shows a varied global spread across the globe. It showed extreme variation ranging from brown, intermediate and blue. The percentage of eye colour for blue, intermediate and brown eye colour differs in different populations. The highest percentage of any colour is observed in Uzbekistan based on the available literature, where more than 90% of the population have brown-coloured eyes. In contrast, brown-coloured eyes are less prevalent in Iceland. Blue eye colour is prevalent in Iceland with nearly three-quarters (74.52%) of the population possessing blue-coloured eyes while the lowest blue eye colour was reported in Iran (1%). Intermediate eye colour is less prevalent, with a maximum prevalence in the Spanish population (55.2%) and a minimum prevalence in Uzbekistan (6.02%) [13]. Genome-wide association studies (GWAS) have revealed that different types of genes are involved in complex traits, such as externally visible characteristics (EVCs) in human beings [14]. Eye and Hair pigmentation in humans is a polygenic trait involving different genes controlling the synthesis and localization of melanin [10]. Strong associations of numerous SNPs have been found with differences in eye and hair colour [10]. In 2014, the HIrisPlex system was released which is used as an EVC prediction tool. This model predicts three EVCs: hair, eye and skin colour. HIrisplex shares six SNPs with Irisplex, a previously developed interactive model [15]. IrisPlex is an eye pigmentation prediction tool developed by the same research group that classifies an individual’s eye colour as being either blue, brown or intermediate based purely upon their DNA [16]. This assay is fully compatible with Scientific Working Group on DNA Analysis Method (SWGDAM) guidelines, who governs forensic assays and validations [15,16].

Pakistan is the sixth-most populous nation in the world with a population of more than 212 million [17]. Pakistan is connected to Central Asia, South Asia and West Asia, and there have been several instances of human migration to this region throughout recorded history. At present, the population of Pakistan is divided into 18 ethnic groups and more than 60 different languages have been reported [18]. The Pakhtoons are the largest ethnic group in the Khyber Pakhtunkhwa province and the second largest ethnic group in Pakistan (15.4%) [19]. For this large population, very limited documentation about forensically informative DNA markers is available so far. Therefore, it is needed to record the eye colour prevalence and also to check the validation of the Irsisplex system on the Pakhtoon population in the Malakand Division. The results of this study may prove beneficial for researchers in the field of population genetics, anthropology and forensic genetics.

## 2. Materials and Methods

### 2.1. Study Area

Malakand Division is situated in KP province of Pakistan. It is linked on the north with Afghanistan to the south with Bajaur and Mohmand agencies, to the south with Mardan, Charsadda and Peshawar districts, and to the east with Gilgit-Baltistan. The total population of Malakand Division is approximately 8.67 million (census of Pakistan, 2017). The Pakhtoon population is dominant in Malakand Division and the scattered population of Kohistanis and Gujjars are also present in Malakand Division. The Yousafzai is a sub-tribe of Pakhtoon, currently living in the Northern parts of Khyber-Pakhtunkhwa, Pakistan.

### 2.2. Eye Colour Digital Photograph Collection

Before photographs collection study criteria were set. A total of 893 healthy females and males aged 11–40 were included in the current study. Individuals having Albinism or heterochromia condition were excluded [20]. A light box was built for photo collection to ensure equal distance and lighting conditions for all photos. Photographs were collected according to the published protocol [19,21].

### 2.3. Eye Colour Classification

Seven trained observers assessed the iris colour. This was accomplished by allowing the observers to evaluate the images under similar lighting conditions and from the same distance. They assigned the iris colour to one of the three categories, i.e., brown, intermediate and blue. The intermediate colour was either green or hazel. In cases where no consensus was reached among observers, the samples were eliminated from the study.

### 2.4. Buccal Swabs Sample Collection and DNA Extraction

Buccal swabs samples were collected from the local resident Pakhtoon population. Genomic DNA was extracted according to the published protocol [18]. The DNA was checked on 0.8% agarose gel.

### 2.5. SNPs Amplification and Genotyping

Six SNPs namely, *HERC2*-rs12913832, *IFR4*-rs12203592, *OCA2*-rs1800407, *SLC24A*4-rs16891982, *SLC45A2*-rs1393350, and *TYR*-rs12896399 genes, were selected from the reported literature [17]. The published protocol was used for SNP’s amplification. Capillary electrophoresis was performed using ABI Prism 3730xl Genetic Analyzer Applied Biosystems, Foster City, California 94404, USAand genotypes were extracted through Gene Mapper software.

### 2.6. Diversity of Eye Colour in the Study Population

In order to measure genetic variance in eye colour, the F-statistics (Fst) were applied to compare Pakhtoon population with previous data from Central South Asian populations (Brahui, Balochi, Hazara, Makrani, Sindi, Pathan, Kalash and Brusho), African (Mbuti), East Asians (Han, Japanese, Yakut, Tujia, Yizu and Mongola), Middle East (Druze and Palastine), Americas (Karitin, Columbia, Surui, Maya and Bantu), Europeans (Russia, Orcadian, Sardinia, French, Basque and Adygi) and Oceania (Melanesi and Guin) [21] using Genepop 4.7.5.Software. The non-metric multidimensional scaling (MDS) was used to compare the diversity in eye colour of Pakhtoon populations with above-mentioned populations using SPSS software.

### 2.7. Statistical Analysis

The genotypes for the six IrisPlex SNPs (rs12913832, rs1800407, rs12896399, rs16891982, rs1393350 and rs12203592), using multinomial logistic regression analyses, association tests were conducted on gender, age categories, districts and eye colour using IBM SPSS Statistics v. 23 (SPSS Inc., Chicago, IL, USA). For this purpose, eye colour was categorized as blue, brown and intermediate. A total of 95% confidence intervals (CI) and, respectively, equal to or less than 0.05 significant *p*-values were used. SNPs genotypes were coded as 0, 1 and 2. Gender was coded as male as (1) and females as 2. Age groups and districts were coded as (1, 2, 3, 4, 5, 6 and 7).

### 2.8. Prediction of Eye Colour

The IrisPlex (http://hirisplex.erasmusmc.nl (accessed on 15 January 2021)) and FROG-Kb (forensic reference on genetics knowledge base) (http://frog.med.yale.edu/FrogKB/FrogServlet (accessed on 10 February 2021)) were used. These models predict eye colour with three possible outcomes (blue, intermediate and brown).

## 3. Results

### 3.1. Categorization of Eye Colour

**Figure 1:**
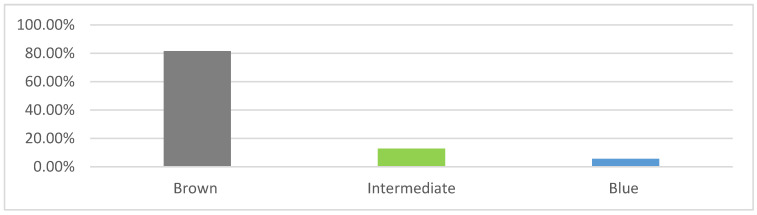
Frequencies of different eye colours.

The present study showed (81.6%) have brown, (12.8%) have intermediate and (5.6%) have blue eye colour (Figure 1).

### 3.2. Eye Colour Based on Gender

**Figure 2:**
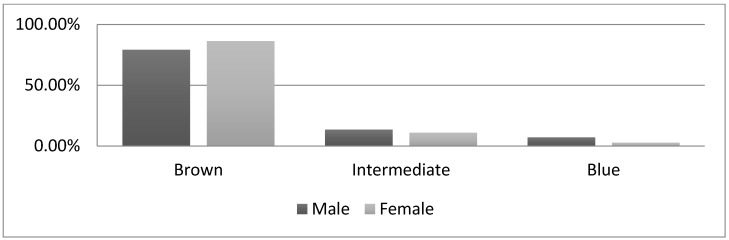
Percentage of eye colour based on gender.

When examining eye colour based on gender, it was discovered that women had a larger percentage of brown eyes, 86.3% (95% CI 0.002–2.277, *p*-value 0.133), than men, who had 79.3% (95% CI 0.074–1.338, *p*-value 0.117).Likewise, intermediate eye colour was higher in males, 13.6%, (95% CI 0.747–13.575, *p*-value 0.135) than in females, 11.00% (95% CI 0.439–500.329 *p*-value 0.175). Blue eye colour was dominant in males, 7.1% (95% CI 0.934–52.002 *p*-value 0.050), than females (2.7%), (95% CI 0.050–10.932 *p*-value 1.000), respectively (Figure 2) (Table 1).

### 3.3. Age Based Eye Colour Classification

**Figure 3:**
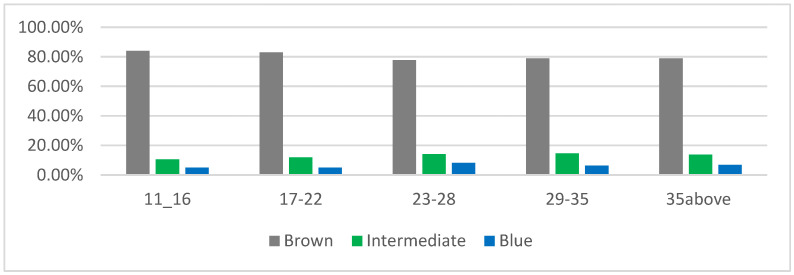
Age-based eye colour prevalence in the study population.

Eye colour was classified into different ages. The frequency of each eye-coloured individual and the significance of the eye colour with age group are shown in (Figure 3) (Table 2).

### 3.4. Genotypes across in the Study Population

Different frequencies of genotypes were observed in the study population; a detail of genotypes in the form of percentages are presented in (Table 3). Details of SNPs are present in (Supplementary Table S1).

After the multinomial logistic test, three variables were found significant with eye colour. The rs12913832 was significant with a brown eye *p*-value of 0.014, an intermediate eye *p*-value of 0.017 and a blue eye colour *p*-value of 0.032. The SNP rs16891982 was also significant with a brown *p*-value of 0.000 and an intermediate *p*-value of 0.000, and SNP rs1393350 with a brown eye *p*-value of 0.015 and an intermediate *p*-value of 0.023 eye colour, while the remaining two SNP, rs1800407 and rs12896399, were non-significant with eye colour in Table 4.

### 3.5. Multinomial Logistic Regression of Eye Colour with Districts

District Swat was significantly associated with *p*-value = (0.050) with brown eye colour and blue *p*-value = (0.012), while district Buner was significant with brown *p*-value = (0.026) and intermediate *p*-value = (0.015), while the rest of the districts (Shangla, Malakand, U. Dir, L. Dir and Chitral) were non-significant Table 5.

### 3.6. Eye Colour Diversity in the World Population

**Figure 4:**
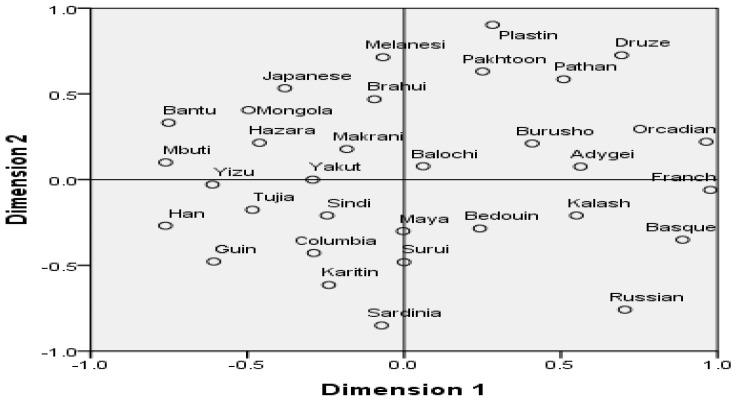
MDS matrix performed with SPSS. The distance among populations is calculated from Fst.

The Fst results were analysed for the six single nucleotide polymorphisms used in the current study The MDS matrix compared the Pakhtoon population to the Pathan population and found some differences between with populations of Palastin, Burusho, Orcadian, Balochi, and Adygei. While the rest of world’s population is far away from the Pakhtoon population in listed in Figure 4.

### 3.7. Eye Colour Prediction Models

#### 3.7.1. Validation of IrisPlex Assay

This study included already published SNPs for the prediction of the eye colour model [15]. The model-based calculator uses prediction probabilities for the three eye colours. Based on the previous study [22], raw probabilities were used, and the category was assigned using the highest probability in each category. For each individual, prediction probabilities were calculated and compared to the already reported eye colour. Of the total 150 tested samples, blue and brown eye colour was predicted accurately in all cases, while intermediate eye colour was predicted as blue 55.55% of the time, and the remaining 45.45% was brown eye colour as shown in Table 6. Total probability values and a record of each eye sample are listed in (Supplementary Table S2).

#### 3.7.2. Validation of FROG-Kb (Forensic Reference on Genetics Knowledge Base)

**Figure 5:**
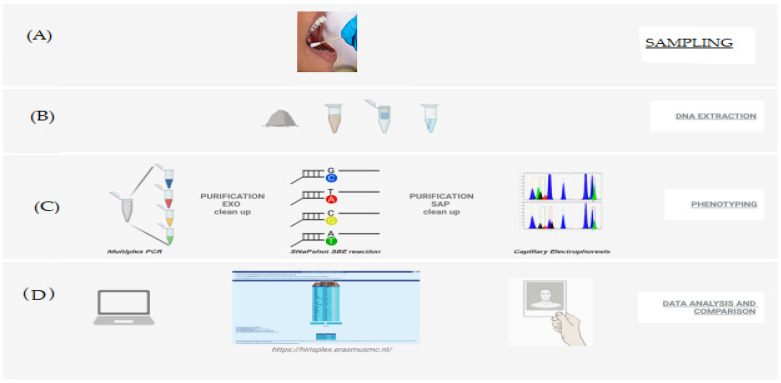
Illustration of the workflow for multiplex PCR phenotyping. Outlining of the main work steps, (**A**) Buccal swab sampling, (**B**) gDNA extraction, (**C**) multiplex PCR, purification of amplicons with exonuclease, SBE reactions, enzymatic purification with SAP, capillary electrophoresis, (**D**) integration of genetic data in the on-line table to obtain phenotypic results, and finally, comparison of these with the photograms of the individuals.

In the current study, a total of 151 samples were checked, in all cases, blue and brown eye colour was predicted accurately, while intermediate eye colour of the individuals was predicted as blue in 53.84% and the rest of the 46.16% of individuals were predicted as brown as shown as in Table 6. Total probability values and a record of each eye sample are listed in (Supplementary Table S3). The workflow of the current study is summarized in Figure 5. 

## 4. Discussion

Eye colour shows a varied global spread across the globe showing extreme variation in the eye colour pigmentation ranging from blue to intermediate to brown [23]. Higher frequencies of brown categories were reported in Azerbaijan, Armenia, Georgia, Kazakhistan, Tajikistan and Uzbekistan [13,24], Korea [25] and Japan [26]. In the current research, we report that people in the Malakand Division tend to have brown eyes as shown in Figure 1. This study is comparable to the studies previously mentioned. This is in contrast to earlier studies that indicated that people with brown eyes make up a very small percentage of the population compared to people with other coloured irises in Iceland, Germany and Denmark [13,27,28,29]. The sampled individuals in the current research revealed that 12.8% had intermediate eye colour. Similar findings were reported previously, for example, in Iceland as 14.15%, in Kazakhstan as 11.65% and in Poland as 12.5% of intermediate eye-coloured individuals [13,23,30]. Tajikistan has a ratio of 7.67% and Uzbekistan has a ratio of 6.02% for intermediate eye tone [24]. In contrast, different intermediate colour frequencies are reported (44%) in France, (39.6%), in Germany (55.2%) and in the Spanish population [12,27]. Blue colours are more prevalent in people of Denmark (64.84%), Poland (52.50%) and Iceland (73.90%) [28,30]. Our study is different from the aforementioned studies but has similar results to those in the populations of Azerbaijan, Armenia, Georgia and Tajikistan [13]. Different populations in European, East Asian, South Asian, African, and American populations displayed different eye colour ratios [31,32,33,34,35], but in general, brown eyes were more prevalent in East Asia, North America, Oceania, Africa, and Sub-Saharan Africa than blue eyes in Northern Europe [36,37,38]. Eye colour has been certainly connected with demographic features, such as sex and age. In the current study, brown eye colour is higher in females, while blue and intermediate is higher in males. When a multinomial regression test was applied, we also discovered a relationship between gender and eye colour. With blue eyes, males had a significant *p*-value of (0.050) in Table 1, but the remainder of the eye colours and gender did not. Similar findings were also found in the populations of Italy and Spain, where men tended to have intermediate and blue eyes while females tended to have brown eyes [39,40]. The same outcomes, though, could not be replicated in the populations of Sweden and Denmark [41]. Consequently, the current and previous results showed that eye colour and sex association are mainly restricted to some specific populations. This significant association confirms that there must be an unknown factor that controls human eye colour variation. A further study is required in order to elucidate the influence of gender on eye colour variation in humans.

A consistent age category has not yet been given, and various researchers have used different categories to classify eye colour [25,26,42,43,44,45,46,47]. In the current study, it was reported that the prevalence of eye colour is based on age groups, and brown eye colour is higher throughout the entire study. Some findings were comparable; with a predominance of brown eyes in different age groups, i.e., Ref. [25] revealed that the majority of people in Korea between the ages of 13 and 80 had brown eyes. A study on Japanese people discovered that the population of Japan has a greater percentage of brown eyes [26]. A research team working on two separate studies, one on a population older than one year and the other on a population older than seven, discovered that brown eyes are most common [46,47]. Some of the results were in contrast to our research [44], which examined 52–93-year old European Australians and discovered that blue/grey eyes are frequently seen. Another research study conducted in Northeast Europe on 12-year-olds and Americans aged 43 to 86 [42], Australians over the age of 49 [43], and Americans aged 60 to 69 [33] revealed that people with blue eyes were more common [33]. Even with the importance of eye colour in age groups, very few studies are available; only one study is significant with brown eye colour in the age groups 31–40 and 60–to above with blue eye colour [47]. Our finding is a contrast where no significance was found between age groups and eye colour in Table 2. The difference between our study and previous ones may be due to age category selection or may be due to ethnic and environmental factors.

In the current study, we found different percentages of the genotypes in specific SNPs when compared worldwide [21,29]. Genotyped of the three SNPs, rs12896399, rs12913832 and rs16891982 have an unevenly inverse genotypes ratio in Northern European when matched with Spain.

Developing a better understanding of eye colour will contribute greatly to the fields of forensics, anthropology and public health. At present, many forensic groups developed pigmentation predictor tools. The primary purpose of these tools is to allow crime scene investigators to predict the eye colour of unidentified individuals from small samples of their DNA. Irisplex and FROG-Kb are perhaps the most well known of these tools. A previous study in an Italian population has shown 76% accuracy of the Irisplex system [48]. A similar study found 95%, 58% and 11% accuracy on the Irisplex system for the prediction of blue, brown and intermediate eye colour, respectively [49]. In the study, 89% accuracy for blue eye colour and 94% for brown eye colour was found [50]. In contrast, the present study reports a 100% accurate prediction for blue and brown eye colour while minority intermediate eye colour phenotypes are misclassified with 23.08% predicted as blue and 76.92% predicted as brown. Intermediate dark phenotypes were mostly predicted as brown, and a light eye-coloured individual was predicted as blue. Moreover, none of the individuals were unclassified. Issues in predicting intermediate eyes have already been shown in previous studies [15,16,18,20,33,51,52,53,54,55,56,57,58,59].

In the current study, we also compared the two models, FROG-kb and HIrisplex, and the same results were obtained. Through FROG-kb, brown eye colour was predicted 100% accurately, similar to the studies [15,16].

Overall, we used phenotype SNPs to obtain eye colour predictions. The comparison of the three prediction systems revealed that IrisPlex and FROG-Kb same higher prediction ratios for blue and brown eye colour. Our results suggest that iris colour SNPs should be analysed for the population in which the system IrisPlex is going to be implemented. Our results also show that the district Swat was significantly associated *p*-value = (0.050) with brown eye colour, blue *p*-value = (0.012), while district Buner was significant with brown *p*-value = (0.026), intermediate *p*-value = (0.015) while the rest of districts (Shangla, Malakand, U. Dir, Chitral and L. Dir) were non-significant Table 5. A case-wise study was performed in Himachal Pradesh, Kangra, where the intermediate eye ratio is higher in a few generations at the temperate climate from Uttar Pradesh, Gorakhpur, Bihar and Darbhanga which are humid subtropical regions. Thus, climate might have a role in eye pigmentation due to the differential expression of the genes responsible for eye colour [60]. Hence, the exact molecular mechanisms needed to be unearthed.

In the present study, the association of the (rs12913832) SNP in HERC2 gene with eye colours in the Pakhtoon Population of the Malakand Division was investigated. The results were similar to some earlier studies [24,29,51,61]. The SNP rs1800407 does not show a significant correlation. However, this SNP rs1800407 was previously found significant with eye colour [50,52]. The significant association of rs12896399 SNP was found with eye colour determination [30,50,54] but, in the present study, this SNP has non-significant. The significance of rs16891982 SNP in eye colour prediction is lower in Northern Europe and higher in Southern Europe populations [30]. The present finding also elucidated the higher significance of this SNP in eye colour prediction. In the present study, a significant association of rs1393350 SNP was found with eye colour, while [30] showed a non-significant association of SNPs rs1393350 with eye colour. The weak association of this rs12203592 SNPs was found with eye colour [55,61,62]. While from the study [39], rs12203592 was strongly associated with eye colour. While in the current study, rs12203592 SNP was excluded from the analysis as it was not polymorphic in our study population.

Khyber Pakhtunkhwa (KPK) formerly called North-West Frontier Province is located in the northwest of Pakistan and has an estimated 13.4% of the total population of Pakistan in which Pakhtuns are the major ethnic group [61]. Furthermore, we examined the effect of the six DNA variants included in the IrisPlex system on their potent ability to infer biogeographic ancestry. It had been advocated before those SNPs from pigmentation genes are useful for genetic ancestry detection [62]. Figure 4 showed a two-dimensional plot of a non-metric multidimensional scaling (MDS) analysis of pairwise FST values and the findings showed that, based on eye colour, the Pakhtoon population of the Malakand Division is unique from other populations around the world. The MDS matrix put the Pakhtoon population at a near distance to the Pathan population and next to the Palastin, Burusho, Orcadian, Balochi and Adygei populations The MDS matrix compared the Pakhtoon population to the Pathan population and found some differences between with populations of Palastin, Burusho, Orcadian, Balochi, and Adygei., while the rest of world’s populations are more different from the Pakhtoon population. However, previous studies based on different molecular markers showed different results. Most of the historians believe that Pakhtoon is the offspring of Jews, and some novelists from Europe believe that Pakhtoon are Caucasian ethnic group that originated from Armenians, some authors also believe that Pakhtoon basically belong to Arians [63]. Other researchers found Cultural and ethnic similarities between Pakhtoon and Jews [4,61]. From an earlier study, molecular ancestors confirmed minor contributions to the Pakhtoon DNA from Arab, Greek, Iranian and Turkish people [64]. The Pakhtoon’s ancestry with different ethnic groups (ethnic groups from Afghanistan, Pakistan and neighbouring countries) were investigated using Y STR loci, and it was confirmed that they have no ties to Israel or the Jewish people [65]. We concluded that the genetic background for iris colour in the Pakhtoon population living in Malakand Division is genetically different from that of other populations of the world. Our results highlight the importance of studying the variation of these genes and their association with iris colour, not only in this population but among the world’s population for identification of people based on iris colour.

## 5. Conclusions

This study concluded that phenotypically in Malakand Division the ratio of brown eye-coloured individuals is high. Multinomial logistic regression analysis between particular variables and eye colour showed a significant *p*-value (<0.05) for rs16891982 SNP, rs12913832 SNP, rs1393350 SNP, districts and gender with eye colour. The rs12896399 SNP, SNP rs1800407 and ages were non-significant with eye colour, respectively. The rs12896399 SNP and SNP rs1800407 were found significant with rs16891982 SNP. We concluded in the current study that the genetic background for iris colour in the Pakhtoon population is genetically different to that of other populations. Importantly, the DNA prediction model (IrisPlex and FROG-Kb) showed a higher accuracy for predicting brown and blue eye colour while uncertainty remained for intermediate eye colour. These findings may encourage the application of the multiplex for the prediction of eye, traits from DNA in forensic cases, particularly in cases where no other identification method can be applied or where chromatic information can support an anthropological investigation. In fact, the FDP shows a very broad spectrum of applications in the forensic field, both in terms of identification based on traces and cadaveric identification in multiple fields (e.g., missing persons, human trafficking and mass disasters). In the future, the implementation of DNA markers related to eye chromatism and their addition to the multiplex system would prove fundamental to improving the prediction accuracies of the “intermediate” categories which with the described model are detected with a much lower accuracy compared to the “brown” and “blue” eye colour categories.

## Figures and Tables

**Table 1:** Multinomial logistic regression analysis between gender and eye colour. The bold value indicated the significance with eye colour.

	Brown Eye Colour				Intermediate Eye Colour				Blue Eye Colour			
Variables	B	Sig.	95% Confidence Interval.		B	Sig.	95%. Confidence Interval		B	Sig.	95% Confidence Interval	
			L. Bound	U. Bound			Lower Bound	U. Bound			L. Bound	U. Bound
Male	1.158	0.117	0.074	1.338	1.158	0.135	0.747	13.575	1.941	0.050	0.934	52.002
Female	2.696	0.133	0.002	2.277	2.696	0.175	0.439	500.329	3.209	1.000	0.050	10.932

**Table 2:** Multinomial logistic regression analysis between age groups and eye colour.

	Brown Eye Colour				Inter Eye Colour				Blue Eye Colour			
Age Groups	B	Sig.	95% Confidence Interval.		B	Sig.	95%. Confidence Interval		B	Sig.	95% Confidence Interval	
			Lower Bound	Upper Bound			Lower Bound	Upper Bound			Lower Bound	Upper Bound
11–16	2.679	0.120	0.002	2.010	2.679	0.120	0.498	426.969	−1.060	0.560	0.010	12.208
17–22	1.711	0.317	0.006	5.163	1.711	0.317	0.194	158.184	−0.303	0.826	0.050	10.932
23–28	1.359	0.404	0.011	6.274	1.359	0.404	0.159	95.083	−1.468	0.337	0.011	4.621
29–34	1.920	0.254	0.005	3.959	1.920	0.254	0.253	184.339	−0.078	0.958	0.051	16.888
35–40	0.558	0.785	0.032	96.100	−0.558	0.785	0.010	31.477	18.240	0.999	0.006	4.601

**Table 3:** Percentage of Irisplex SNPs present in Pakhtoon population.

	Genotypes							
SNPs	CT	TT	CC	GG	GA	CA	GC	AA
rs12913832	49.40%	31.60%	19.00%	-	-	-	-	-
rs1800407	-	-	-	82.30%	17.70%	-	-	-
rs12896399	-	-	58.20%	-	-	35.50%	-	6.30%
rs16891982	-	-	15.20%	51.90%	-	-	32.90%	
rs1393350	-	-	-	87.30%	11.40%	-	-	1.30%
rs12203592	-	-	100%	-	-	-	-	-

**Table 4:** Result of Multinomial logistic regression between SNPs and eye colour. The bold value indicated the significant with eye colour.

Variables	Brown Eye Colour				Intermediate Eye Colour				Blue Eye Colour			
	B	Sig.	95% Confidence Interval.		B	Sig.	95%. Confidence Interval		B	Sig.	95% Confidence Interval	
			L Bound	U Bound			L Bound	U Bound			L Bound	U Bound
rs12913832	1.458	**0** **.014**	1.348	13.702	1.458	**0** **.017**	0.073	1.530	−0.417	**0** **.032**	0.284	1.530
rs1800407	1.729	0.113	0.664	47.810	1.729	0.113	0.021	7.670 × 10^−9^	18.686	0.211	7.670 × 10^−9^	7.670 × 10^−9^
rs12896399	0.203	0.597	0.578	2.595	−0.203	0.597	0.385	1.837	−0.588	0.335	0.168	1.837
rs16891982	2.302	**0** **.000**	3.137	31.829	2.302	**0** **.000**	0.031	106.849	1.866	0.192	0.391	106.849
rs1393350	2.054	0.015	1.484	41.000	2.054	0.023	0.024	24.597	16.342	0.998	0.011	6.274

**Table 5:** Result of Multinomial logistic regression analysis between Districts and eye colour. The bold value indicated significant variable with eye colour.

	Brown Eye Colour				Intermediate Eye Colour				Blue Eye Colour			
Districts	B	Sig.	95% Confidence Interval.		B	Sig.	95%. Confidence Interval		B	Sig.	95% Confidence Interval	
			L. Bound	Upper Bound			Lower Bound	Upper Bound			Lower Bound	Upper Bound
Malakand	0.270	0.857	0.070	24.597	−0.270	0.857	0.041	14.338	−0.196	0.888	0.053	12.718
Swat	2.392	**0** **.050**	0.007	1.129	2.392	0.062	0.886	134.960	2.863	**0** **.012**	1.855	165.339
Buner	3.082	**0** **.026**	0.003	0.686	3.082	**0** **.015**	1.458	326.260	1.370	0.356	0.214	72.363
Shangla	1.769	0.293	0.217	158.156	1.769	0.273	0.006	4.601	1.286	0.411	0.169	77.455
L. Dir	1.533	0.269	0.014	3.266	1.533	0.229	0.306	70.006	0.626	0.568	0.217	16.106
Chitral	0.313	0.856	0.046	40.755	−0.313	0.756	0.025	21.784	18.893	0.998	0.385	1.837
U. Dir	−0.312	1.000	0.003	0.686	0.312	1.000	0.070	24.597	1.999	1.000	0.007	1.129

**Table 6:** Eye colour prediction model validation: Bold values show successful prediction estimates from the local resident Pakhtoon population.

		Predicted Eye Colour				
Prediction Models	Eye Colour	Blue	Brown	Intermediate	Undetected	No of Samples
**IrisPlex**	**Blue**	**100%**	0	0	0	(13)
	**Brown**	0	100%	0	0	(111)
	**Intermediate**	**55.55%**	**45.45%**	0	0	(26)
**FROG-kb**	**Blue**	**100%**	0	0	0	(13)
	**Brown**	0	100%	0	0	(111)
	**Intermediate**	**53.84%**	**46.16%**	0	0	(26)

## Data Availability

All the relevant data is available within the manuscript and its Supplementary Materials.

## Supplementary Materials

The following supporting information can be downloaded at: https://www.mdpi.com/article/10.3390/biomedicines11041228/s1, Table S1: SNPs Genotype Profiles, Table S2: Pakhtoon population sample details and their Irisplex SNP genotypes prediction details. Table S3: Pakhtoon population sample details and their FROG-kb SNP genotypes prediction details. Green indicated Intermediate eye colour.
