# Supplementary material for: Phenotypic Classification of Eye Colour and Developmental Validation of the Irisplex System on Population Living in Malakand Division, Pakistan
Source: Biomedicines. 2023 Apr 20;11(4):1228. doi: 10.3390/biomedicines11041228 (PMC10136328; doi:10.3390/biomedicines11041228)
Supplement: Supplementary file 1 [file biomedicines-11-01228-s001.zip › biomedicines-2322476-supplementary.pdf]

**Supplementary Materials:**

Table S 1: SNPs Genotype Profiles

| Sample | rs12913832 | rs1800407 | rs12896399 | rs16891982 | rs1393350 | rs12203592 |
|---|---|---|---|---|---|---|
| DNA1 | CT | GG | CC | GC | GG | CC |
| DNA2 | TT | GA | AA | GC | GG | CC |
| DNA3 | CT | GG | AA | GC | AA | CC |
| DNA4 | TT | GG | CA | CC | GA | CC |
| DNA5 | CC | GG | CC | GG | GG | CC |
| DNA6 | TT | GA | CA | CC | GG | CC |
| DNA7 | CC | GG | CA | GC | GG | CC |
| DNA8 | CC | GG | CC | GC | GG | CC |
| DNA9 | CC | GG | CC | GC | GG | CC |
| DNA10 | TT | GG | CA | GG | GG | CC |
| DNA11 | CC | GG | AA | CC | GA | CC |
| DNA12 | CC | GG | CA | CC | GG | CC |
| DNA13 | CC | GG | CA | CC | GG | CC |
| DNA14 | TT | GG | CC | GG | GG | CC |
| DNA15 | CC | GG | CA | CC | GG | CC |
| DNA16 | CC | GG | AA | GC | GG | CC |
| DNA17 | CC | GG | CC | GC | GG | CC |
| DNA18 | TT | GG | CA | GG | GG | CC |
| DNA19 | CC | GG | CC | GG | GG | CC |
| DNA20 | CC | GG | CC | GC | GG | CC |
| DNA21 | TT | GG | CC | GG | GG | CC |
| DNA22 | CT | GG | CA | GG | GG | CC |
| DNA23 | CT | GG | CA | GC | GG | CC |
| DNA24 | TT | GA | CC | GG | GG | CC |
| DNA25 | TT | GG | CA | GC | GG | CC |
| DNA26 | TT | GG | CA | GG | GG | CC |
| DNA27 | CC | GG | CC | GG | GG | CC |
| DNA28 | CT | GG | CC | GC | GA | CC |
| DNA29 | TT | GG | CC | GG | GA | CC |
| DNA30 | TT | GG | CC | GG | GG | CC |
| DNA31 | CT | GG | CC | GG | GG | CC |
| DNA32 | CT | GG | CC | GG | GG | CC |
| DNA33 | CT | GG | CA | GC | GG | CC |
| DNA34 | CT | GG | CA | GG | GG | CC |

| | | | | | | |
|---|---|---|---|---|---|---|
| DNA35 | CT | GG | CC | GG | GG | CC |
| DNA36 | CT | GG | CC | GG | GG | CC |
| DNA37 | CT | GG | CC | GG | GG | CC |
| DNA38 | CT | GG | CC | GC | GG | CC |
| DNA39 | CT | GG | CA | GG | GG | CC |
| DNA40 | CT | GG | CC | GC | GG | CC |
| DNA41 | TT | GA | CC | GG | GA | CC |
| DNA42 | CT | GG | CC | GG | GG | CC |
| DNA43 | TT | GG | CA | GG | GG | CC |
| DNA44 | TT | GG | CC | GG | GG | CC |
| DNA45 | CT | GG | AA | GG | GG | CC |
| DNA46 | CC | GG | CC | GG | GG | CC |
| DNA47 | CT | GA | CC | GC | GG | CC |
| DNA48 | CT | GG | CC | GC | GA | CC |
| DNA49 | CT | GG | CC | GC | GG | CC |
| DNA50 | TT | GG | CA | GC | GA | CC |
| DNA51 | CT | GG | CA | GG | GG | CC |
| DNA52 | CT | GA | CC | GC | GG | CC |
| DNA53 | TT | GG | CC | GG | GG | CC |
| DNA54 | CT | GA | CC | GC | GA | CC |
| DNA55 | TT | GG | CA | GG | GG | CC |
| DNA56 | CT | GG | CA | GG | GG | CC |
| DNA57 | TT | GA | CA | GG | GG | CC |
| DNA58 | CT | GG | CA | GG | GG | CC |
| DNA59 | 0 | 0 | 0 | 0 | 0 | 0 |
| DNA60 | CT | GG | CC | GG | GG | CC |
| DNA61 | CT | GG | CA | GC | GG | CC |
| DNA62 | TT | GA | CC | GG | GG | CC |
| DNA63 | CT | GG | CA | GC | GG | CT |
| DNA64 | CT | GG | CC | CC | GG | CC |
| DNA65 | CT | GA | CA | CC | GG | CC |
| DNA66 | CT | GA | CC | CC | GG | CC |
| DNA67 | TT | GA | CC | CC | GG | CC |
| DNA68 | CT | GG | CC | GG | GG | CC |
| DNA69 | TT | GA | CA | GC | GG | CC |
| DNA70 | CC | GG | CC | GG | GG | CC |
| DNA71 | TT | GA | CA | GG | GA | CC |
| DNA72 | TT | GG | CC | GG | GG | CC |

| | | | | | | |
|---|---|---|---|---|---|---|
| DNA73 | CT | GG | CC | GG | GG | CC |
| DNA74 | TT | GG | CC | GC | GG | CC |
| DNA75 | CT | GG | CA | CC | GG | CC |
| DNA76 | CT | GG | CC | GC | GG | CC |
| DNA77 | TT | GG | CC | GG | GG | CC |
| DNA78 | CT | GG | CC | GG | GG | CC |
| DNA79 | CT | GG | CC | GG | GG | CC |
| DNA80 | CT | GG | CC | CC | GG | CC |
| DNA81 | TT | GG | CC | GG | GG | CC |
| DNA82 | TT | GG | CC | GG | GG | CC |
| DNA83 | CT | GG | CC | GG | GG | CC |
| DNA84 | CC | GG | CC | GC | GG | CC |
| DNA85 | CT | GG | CC | GG | GG | CC |
| DNA86 | CT | GG | AA | GC | AA | CC |
| DNA87 | TT | GG | CC | GG | GG | CC |
| DNA88 | CT | GG | AA | GC | AA | CC |
| DNA89 | CC | GG | CC | GG | GG | CC |
| DNA90 | CT | GG | AA | GC | AA | CC |
| DNA91 | CT | GG | CC | GG | GG | CC |
| DNA92 | CT | GG | CC | GG | GG | CC |
| DNA93 | TT | GG | CC | GG | GG | CC |
| DNA94 | TT | GG | CA | GG | GG | CC |
| DNA95 | CC | GG | CC | GG | GG | CC |
| DNA96 | CT | GG | CC | GG | GG | CC |
| DNA97 | TT | GG | CA | GG | GG | CC |
| DNA98 | TT | GG | CC | GG | GG | CC |
| DNA99 | TT | GG | CA | GG | GG | CC |
| DNA100 | CC | GG | CA | CC | GG | CC |
| DNA101 | CT | GG | CC | GG | GG | CC |
| DNA102 | CT | GG | AA | GC | AA | CC |
| DNA103 | TT | GG | CC | GG | GG | CC |
| DNA104 | TT | GG | CA | GG | GG | CC |
| DNA105 | CT | GG | CC | GG | GG | CC |
| DNA106 | TT | GG | CC | GG | GG | CC |
| DNA107 | CT | GG | AA | GC | AA | CC |
| DNA108 | CT | GG | CC | GG | GG | CC |
| DNA109 | TT | GG | CC | GG | GG | CC |
| DNA110 | TT | GG | CC | GG | GG | CC |

| | | | | | | |
|---|---|---|---|---|---|---|
| DNA111 | CT | GG | AA | GC | AA | CC |
| DNA112 | TT | GG | CC | GG | GG | CC |
| DNA113 | CT | GG | CC | GG | GG | CC |
| DNA114 | TT | GG | CC | GG | GG | CC |
| DNA115 | TT | GG | CC | GG | GG | CC |
| DNA116 | CT | GG | CC | GG | GG | CC |
| DNA117 | TT | GG | CC | GG | GG | CC |
| DNA118 | CT | GG | AA | GC | AA | CC |
| DNA119 | CC | GG | CA | CC | GG | CC |
| DNA120 | CT | GG | CC | GG | GG | CC |
| DNA121 | TT | GG | CC | GG | GG | CC |
| DNA122 | TT | GG | CA | GG | GG | CC |
| DNA123 | TT | GG | CC | GG | GG | CC |
| DNA124 | TT | GG | CA | GG | GG | CC |
| DNA125 | TT | GG | CC | GG | GG | CC |
| DNA126 | TT | GG | CC | GG | GG | CC |
| DNA127 | TT | GG | CC | GG | GG | CC |
| DNA128 | TT | GG | CC | GG | GG | CC |
| DNA129 | CC | GG | CC | GG | GG | CC |
| DNA130 | TT | GG | CC | GG | GG | CC |
| DNA131 | CT | GG | CC | GG | GG | CC |
| DNA132 | TT | GG | CC | GG | GG | CC |
| DNA133 | CT | GG | CC | GG | GG | CC |
| DNA134 | CC | GG | CC | GG | GG | CC |
| DNA135 | CC | GG | CA | CC | GG | CC |
| DNA136 | CT | GG | CC | GG | GG | CC |
| DNA137 | CC | GG | CC | GG | GG | CC |
| DNA138 | CC | GG | CA | CC | GG | CC |
| DNA139 | TT | GG | CC | GG | GG | CC |
| DNA140 | TT | GG | CC | GG | GG | CC |
| DNA141 | CT | GG | CC | GG | GG | CC |
| DNA142 | CT | GG | AA | GC | AA | CC |
| DNA143 | TT | GG | CC | GG | GG | CC |
| DNA144 | CT | GG | AA | GC | AA | CC |
| DNA145 | TT | GG | CC | GG | GG | CC |
| DNA146 | TT | GG | CC | GG | GG | CC |
| DNA147 | CT | GG | CC | GG | GG | CC |
| DNA148 | TT | GG | CC | GG | GG | CC |

| | | | | | | |
|---|---|---|---|---|---|---|
| DNA149 | CC | GG | CC | GG | GG | CC |
| DNA150 | CC | GG | CC | GG | GG | CC |

Table S 2: Irisplex 6 SNP genotypes colour prediction probabilities together with samples details of Pakhtoon population.

| Sample | rs12913832 | rs1800407 | rs12896399 | rs16891982 | rs1393350 | rs12203592 | P Blue Eye | P Inter Eye | P Brown Eye | Gender | Irisplex Result | Pheno Eye Colour |
|---|---|---|---|---|---|---|---|---|---|---|---|---|
| Swat 1 | CT | GG | CC | GC | GG | CC | 0.01223 | 0.050034 | **0.937736** | M | **Brown** | **Brown** |
| 2 | TT | GA | AA | GC | GG | CC | 0.000223 | 0.013989 | **0.985788** | M | **Brown** | **Brown** |
| 3 | CT | GG | AA | GC | AA | CC | 0.01223 | 0.050034 | **0.937736** | F | **Brown** | **Brown** |
| 4 | TT | GG | CA | CC | GA | CC | 1.29E-05 | 0.002091 | **0.997896** | F | **Brown** | **Brown** |
| 5 | CC | GG | CC | GG | GG | CC | **0.84781** | 0.087663 | 0.064527 | F | **Blue** | **Blue** |
| 6 | TT | GA | CA | CC | GG | CC | 4.87E-05 | 0.005544 | **0.994407** | F | **Brown** | **Brown** |
| 7 | CC | GG | CA | GC | GG | CC | **0.649894** | 0.121839 | 0.228267 | M | **Blue** | **Blue** |
| 8 | CC | GG | CC | GC | GG | CC | **0.649894** | 0.121839 | 0.228267 | F | **Blue** | **INTER** |
| 9 | CC | GG | CC | GC | GG | CC | **0.649894** | 0.121839 | 0.228267 | M | **Blue** | **INTER** |
| 10 | TT | GG | CA | GG | GG | CC | 0.000272 | 0.01339 | **0.986338** | F | **Brown** | **Brown** |
| 11 | CC | GG | AA | CC | GA | CC | 0.337744 | 0.114804 | **0.547452** | F | **Brown** | **INTER** |
| 12 | CC | GG | CA | CC | GG | CC | 0.337744 | 0.114804 | **0.547452** | M | **Brown** | **INTER** |
| 13 | CC | GG | CA | CC | GG | CC | 0.337744 | 0.114804 | **0.547452** | F | **Brown** | **INTER** |
| 14 | TT | GG | CC | GG | GG | CC | 0.000 | 0.0133 | **0.98633** | M | **Brown** | **Brown** |

| | | | | | | | | | | | | |
|---|---|---|---|---|---|---|---|---|---|---|---|---|
| | | | | | | | 272 | 9 | **8** | | | |
| 15 | CC | GG | CA | CC | GG | CC | 0.337744 | 0.114804 | **0.547452** | F | **Brown** | **INTER** |
| 16 | CC | GG | AA | GC | GG | CC | **0.649894** | 0.121839 | 0.228267 | M | **Blue** | **INTER** |
| 17 | CC | GG | CC | GC | GG | CC | **0.649894** | 0.121839 | 0.228267 | M | **Blue** | **INTER** |
| 18 | TT | GG | CA | GG | GG | CC | 0.000272 | 0.01339 | **0.986338** | F | **Brown** | **Brown** |
| 19 | CC | GG | CC | GG | GG | CC | **0.84781** | 0.087663 | 0.064527 | F | **Blue** | **Blue** |
| 20 | CC | GG | CC | GC | GG | CC | **0.649894** | 0.121839 | 0.228267 | M | **Blue** | **INTER** |
| Malakand21 | TT | GG | CC | GG | GG | CC | 0.000272 | 0.01339 | **0.986338** | M | **BROWN** | **BROWN** |
| 22 | CT | GG | CA | GG | GG | CC | 0.050323 | 0.113551 | **0.836126** | M | **BROWN** | **BROWN** |
| 23 | CT | GG | CA | GC | GG | CC | 0.01223 | 0.050034 | **0.937736** | M | **BROWN** | **BROWN** |
| 24 | TT | GA | CC | GG | GG | CC | 0.001006 | 0.034825 | **0.964169** | M | **BROWN** | **BROWN** |
| 25 | TT | GG | CA | GC | GG | CC | 5.94E-05 | 0.005305 | **0.994635** | M | **BROWN** | **BROWN** |
| 26 | TT | GG | CA | GG | GG | CC | 0.000272 | 0.01339 | **0.986338** | M | **BROWN** | **BROWN** |
| 27 | CC | GG | CC | GG | GG | CC | **0.84781** | 0.087663 | 0.064527 | M | **BLUE** | **BLUE** |
| 28 | CT | GG | CC | GC | GA | CC | 0.01223 | 0.050034 | **0.937736** | F | **BROWN** | **BROWN** |
| 29 | TT | GG | CC | GG | GA | CC | 0.000272 | 0.01339 | **0.986338** | M | **BROWN** | **BROWN** |
| 30 | TT | GG | CC | GG | GG | CC | 0.000272 | 0.01339 | **0.986338** | F | **BROWN** | **BROWN** |
| 31 | CT | GG | CC | GG | GG | CC | 0.050323 | 0.113551 | **0.836126** | F | **BROWN** | **BROWN** |
| 32 | CT | GG | CC | GG | GG | CC | 0.050323 | 0.113551 | **0.836126** | F | **BROWN** | **BROWN** |

| | | | | | | | | | | | | |
|---|---|---|---|---|---|---|---|---|---|---|---|---|
| 33 | CT | GG | CA | GC | GG | CC | 0.01223 | 0.050034 | **0.937736** | F | **BROWN** | **BROWN** |
| 34 | CT | GG | CA | GG | GG | CC | 0.050323 | 0.113551 | **0.836126** | M | **BROWN** | **BROWN** |
| 35 | CT | GG | CC | GG | GG | CC | 0.050323 | 0.113551 | **0.836126** | F | **BROWN** | **BROWN** |
| 36 | CT | GG | CC | GG | GG | CC | 0.050323 | 0.113551 | **0.836126** | M | **BROWN** | **INTER** |
| 37 | CT | GG | CC | GG | GG | CC | 0.050323 | 0.113551 | **0.836126** | F | **BROWN** | **BROWN** |
| 38 | CT | GG | CC | GC | GG | CC | 0.01223 | 0.050034 | **0.937736** | F | **BROWN** | **BROWN** |
| 39 | CT | GG | CA | GG | GG | CC | 0.050323 | 0.113551 | **0.836126** | F | **BROWN** | **BROWN** |
| 40 | CT | GG | CC | GC | GG | CC | 0.01223 | 0.050034 | **0.937736** | F | **BROWN** | **INTER** |
| Buner 41 | TT | GA | CC | GG | GA | CC | 0.001006 | 0.034825 | **0.964169** | F | **BROWN** | **BROWN** |
| 42 | CT | GG | CC | GG | GG | CC | 0.050323 | 0.113551 | **0.836126** | F | **BROWN** | **BROWN** |
| 43 | TT | GG | CA | GG | GG | CC | 0.000272 | 0.01339 | **0.986338** | F | **BROWN** | **BROWN** |
| 44 | TT | GG | CC | GG | GG | CC | 0.000272 | 0.01339 | **0.986338** | F | **BROWN** | **BROWN** |
| 45 | CT | GG | AA | GG | GG | CC | 0.050323 | 0.113551 | **0.836126** | F | **BROWN** | **INTER** |
| 46 | CC | GG | CC | GG | GG | CC | **0.84781** | 0.087663 | 0.064527 | M | **BLUE** | **BLUE** |
| 47 | CT | GA | CC | GC | GG | CC | 0.04143 | 0.119162 | **0.839408** | F | **BROWN** | **BROWN** |
| 48 | CT | GG | CC | GC | GA | CC | 0.01223 | 0.050034 | **0.937736** | M | **BROWN** | **BROWN** |
| 49 | CT | GG | CC | GC | GG | CC | 0.01223 | 0.050034 | **0.937736** | M | **BROWN** | **INTER** |
| 50 | TT | GG | CA | GC | GA | CC | 5.94E-05 | 0.005305 | **0.994635** | M | **BROWN** | **BROWN** |
| 51 | CT | GG | CA | GG | GG | CC | 0.050323 | 0.113551 | **0.836126** | M | **BROWN** | **BROWN** |

| | | | | | | | | | | | | |
|---|---|---|---|---|---|---|---|---|---|---|---|---|
| 52 | CT | GA | CC | GC | GG | CC | 0.041 43 | 0.1191 62 | **0.83940 8** | M | **BROW N** | **INTER** |
| 53 | TT | GG | CC | GG | GG | CC | 0.000 272 | 0.0133 9 | **0.98633 8** | F | **BROW N** | **BROWN** |
| 54 | CT | GA | CC | GC | GA | CC | 0.041 43 | 0.1191 62 | **0.83940 8** | M | **BROW N** | **INTER** |
| 55 | TT | GG | CA | GG | GG | CC | 0.000 272 | 0.0133 9 | **0.98633 8** | F | **BROW N** | **BROWN** |
| 56 | CT | GG | CA | GG | GG | CC | 0.050 323 | 0.1135 51 | **0.83612 6** | M | **BROW N** | **INTER** |
| 57 | CT | GA | CA | GG | GG | CC | 0.001 006 | 0.0348 25 | **0.96416 9** | M | **BROW N** | **INTER** |
| 58 | CT | GG | CA | GG | GG | CC | 0.050 323 | 0.1135 51 | **0.83612 6** | M | **BROW N** | **BROWN** |
| 59 | TT | GG | CC | GG | GG | CC | 0.000 272 | 0.0133 9 | **0.98633 8** | F | **BROW N** | **BROWN** |
| 60 | CT | GG | CC | GG | GG | CC | 0.050 323 | 0.1135 51 | **0.83612 6** | F | **BROW N** | **INTER** |
| Shan gla 61 | CT | GG | CA | GC | GG | CC | 0.012 23 | 0.0500 34 | **0.93773 6** | F | **BROW N** | **INTER** |
| 62 | TT | GA | CC | GG | GG | CC | 0.001 006 | 0.0348 25 | **0.96416 9** | F | **BROW N** | **BROWN** |
| 63 | CT | GG | CA | GC | GG | CT | 0.022 247 | 0.0903 19 | **0.88743 4** | M | **BROW N** | **BROWN** |
| 64 | CT | GG | CC | CC | GG | CC | 0.002 76 | 0.0204 76 | **0.97676 4** | F | **BROW N** | **BROWN** |
| 65 | CT | GA | CA | CC | GG | CC | 0.010 029 | 0.0522 98 | **0.93767 4** | F | **BROW N** | **BROWN** |
| 66 | CT | GA | CC | CC | GG | CC | 0.010 029 | 0.0522 98 | **0.93767 4** | M | **BROW N** | **BROWN** |
| 67 | TT | GA | CC | CC | GG | CC | 4.87E-05 | 0.0055 44 | **0.99440 7** | M | **BROW N** | **BROWN** |
| 68 | CT | GG | CC | GG | GG | CC | 0.050 323 | 0.1135 51 | **0.83612 6** | F | **BROW N** | **BROWN** |
| 69 | TT | GA | CA | GC | GG | CC | 0.000 223 | 0.0139 89 | **0.98578 8** | F | **BROW N** | **BROWN** |
| 70 | CC | GG | CC | GG | GG | CC | **0.847** | 0.0876 | 0.06452 | M | **BLUE** | **BLUE** |

| | | | | | | | | | | | | |
|---|---|---|---|---|---|---|---|---|---|---|---|---|
| | | | | | | | **81** | 63 | 7 | | | |
| 71 | TT | GA | CA | GG | GA | CC | 0.001<br>006 | 0.0348<br>25 | **0.96416<br>9** | F | **BROW<br>N** | **BROWN** |
| 72 | TT | GG | CC | GG | GG | CC | 0.000<br>272 | 0.0133<br>9 | **0.98633<br>8** | F | **BROW<br>N** | **BROWN** |
| 73 | CT | GG | CC | GG | GG | CC | 0.050<br>323 | 0.1135<br>51 | **0.83612<br>6** | F | **BROW<br>N** | **BROWN** |
| 74 | TT | GG | CC | GC | GG | CC | 5.94E-<br>05 | 0.0053<br>05 | **0.99463<br>5** | F | **BROW<br>N** | **BROWN** |
| 75 | CT | GG | CA | CC | GG | CC | 0.002<br>76 | 0.0204<br>76 | **0.97676<br>4** | F | **BROW<br>N** | **BROWN** |
| 76 | CT | GG | CC | GC | GG | CC | 0.012<br>23 | 0.0500<br>34 | **0.93773<br>6** | F | **BROW<br>N** | **BROWN** |
| 77 | TT | GG | CC | GG | GG | CC | 0.000<br>272 | 0.0133<br>9 | **0.98633<br>8** | F | **BROW<br>N** | **BROWN** |
| 78 | CT | GG | CC | GG | GG | CC | 0.050<br>323 | 0.1135<br>51 | **0.83612<br>6** | F | **BROW<br>N** | **BROWN** |
| 79 | CT | GG | CC | GG | GG | CC | 0.050<br>323 | 0.1135<br>51 | **0.83612<br>6** | F | **BROW<br>N** | **INTER** |
| 80 | CT | GG | CC | CC | GG | CC | 0.050<br>323 | 0.1135<br>51 | **0.83612<br>6** | F | **BROW<br>N** | **BROWN** |
| L<br>Dir<br>81 | TT | GG | CC | GG | GG | CC | 0.000<br>272 | 0.0133<br>9 | **0.98633<br>8** | M | **BROW<br>N** | **BROWN** |
| 82 | TT | GG | CC | GG | GG | CC | 0.000<br>272 | 0.0133<br>9 | **0.98633<br>8** | M | **BROW<br>N** | **BROWN** |
| 83 | CT | GG | CC | GG | GG | CC | 0.050<br>323 | 0.1135<br>51 | **0.83612<br>6** | F | **BROW<br>N** | **BROWN** |
| 84 | CC | GG | CC | GC | GG | CC | **0.649<br>894** | 0.1218<br>39 | 0.22826<br>7 | M | **BLUE** | **INTER** |
| 85 | CT | GG | CC | GG | GG | CC | 0.050<br>323 | 0.1135<br>51 | **0.83612<br>6** | M | **BROW<br>N** | **BROWN** |
| 86 | CT | GG | AA | GC | AA | CC | 0.012<br>23 | 0.0500<br>34 | **0.93773<br>6** | F | **BROW<br>N** | **BROWN** |
| 87 | TT | GG | CC | GG | GG | CC | 0.000<br>272 | 0.0133<br>9 | **0.98633<br>8** | M | **BROW<br>N** | **BROWN** |
| 88 | CT | GG | AA | GC | AA | CC | 0.012<br>23 | 0.0500<br>34 | **0.93773<br>6** | M | **BROW<br>N** | **BROWN** |

| | | | | | | | | | | | | |
|---|---|---|---|---|---|---|---|---|---|---|---|---|
| 89 | CC | GG | CC | GG | GG | CC | **0.84781** | 0.087663 | 0.064527 | M | **BLUE** | **BLUE** |
| 90 | CT | GG | AA | GC | AA | CC | 0.01223 | 0.050034 | **0.937736** | F | **BROWN** | **BROWN** |
| 91 | CT | GG | CC | GG | GG | CC | 0.050323 | 0.113551 | **0.836126** | F | **BROWN** | **BROWN** |
| 92 | CT | GG | CC | GG | GG | CC | 0.050323 | 0.113551 | **0.836126** | M | **BROWN** | **INTER** |
| 93 | TT | GG | CC | GG | GG | CC | 0.000272 | 0.01339 | **0.986338** | M | **BROWN** | **BROWN** |
| 94 | TT | GG | CA | GG | GG | CC | 0.000272 | 0.01339 | **0.986338** | F | **BROWN** | **BROWN** |
| 95 | CC | GG | CC | GG | GG | CC | **0.84781** | 0.087663 | 0.064527 | M | **BLUE** | **BLUE** |
| 96 | CT | GG | CC | GG | GG | CC | 0.050323 | 0.113551 | **0.836126** | M | **BROWN** | **BROWN** |
| 97 | TT | GG | CA | GG | GG | CC | 0.000272 | 0.01339 | **0.986338** | M | **BROWN** | **BROWN** |
| 98 | TT | GG | CC | GG | GG | CC | 0.000272 | 0.01339 | **0.986338** | F | **BROWN** | **BROWN** |
| 99 | TT | GG | CA | GG | GG | CC | 0.000272 | 0.01339 | **0.986338** | F | **BROWN** | **BROWN** |
| 100 | CC | GG | CA | CC | GG | CC | 0.337744 | 0.114804 | **0.547452** | M | **BROWN** | **INTER** |
| Chitral 101 | CT | GG | CC | GG | GG | CC | 0.050323 | 0.113551 | **0.836126** | M | **BROWN** | **BROWN** |
| 102 | CT | GG | AA | GC | AA | CC | 0.01223 | 0.050034 | **0.937736** | F | **BROWN** | **BROWN** |
| 103 | TT | GG | CC | GG | GG | CC | 0.000272 | 0.01339 | **0.986338** | M | **BROWN** | **BROWN** |
| 104 | TT | GG | CA | GG | GG | CC | 0.000272 | 0.01339 | **0.986338** | F | **BROWN** | **BROWN** |
| 105 | CT | GG | CC | GG | GG | CC | 0.050323 | 0.113551 | **0.836126** | F | **BROWN** | **BROWN** |
| 106 | TT | GG | CC | GG | GG | CC | 0.000272 | 0.01339 | **0.986338** | F | **BROWN** | **BROWN** |
| 107 | CT | GG | AA | GC | AA | CC | 0.012 | 0.0500 | **0.93773** | F | **BROW** | **BROWN** |

| | | | | | | | | | | | | |
|---|---|---|---|---|---|---|---|---|---|---|---|---|
| | | | | | | | 23 | 34 | **6** | | **N** | |
| 108 | CT | GG | CC | GG | GG | CC | 0.050 323 | 0.1135 51 | **0.83612 6** | F | **BROW N** | **BROWN** |
| 109 | TT | GG | CC | GG | GG | CC | 0.000 272 | 0.0133 9 | **0.98633 8** | F | **BROW N** | **BROWN** |
| 110 | TT | GG | CC | GG | GG | CC | 0.000 272 | 0.0133 9 | **0.98633 8** | F | **BROW N** | **BROWN** |
| 111 | CT | GG | AA | GC | AA | CC | 0.012 23 | 0.0500 34 | **0.93773 6** | M | **BROW N** | **BROWN** |
| 112 | TT | GG | CC | GG | GG | CC | 0.000 272 | 0.0133 9 | **0.98633 8** | M | **BROW N** | **BROWN** |
| 113 | CT | GG | CC | GG | GG | CC | 0.050 323 | 0.1135 51 | **0.83612 6** | M | **BROW N** | **BROWN** |
| 114 | TT | GG | CC | GG | GG | CC | 0.000 272 | 0.0133 9 | **0.98633 8** | M | **BROW N** | **BROWN** |
| 115 | TT | GG | CC | GG | GG | CC | 0.000 272 | 0.0133 9 | **0.98633 8** | M | **BROW N** | **BROWN** |
| 116 | CT | GG | CC | GG | GG | CC | 0.050 323 | 0.1135 51 | **0.83612 6** | M | **BROW N** | **BROWN** |
| 117 | TT | GG | CC | GG | GG | CC | 0.000 272 | 0.0133 9 | **0.98633 8** | M | **BROW N** | **BROWN** |
| 118 | CT | GG | AA | GC | AA | CC | 0.012 23 | 0.0500 34 | **0.93773 6** | M | **BROW N** | **BROWN** |
| 119 | CC | GG | CA | CC | GG | CC | 0.337 744 | 0.1148 04 | **0.54745 2** | M | **BROW N** | **INTER** |
| 120 | CT | GG | CC | GG | GG | CC | 0.050 323 | 0.1135 51 | **0.83612 6** | M | **BROW N** | **BROWN** |
| U.Di r121 | TT | GG | CC | GG | GG | CC | 0.000 272 | 0.0133 9 | **0.98633 8** | F | **BROW N** | **BRWON** |
| 122 | TT | GG | CA | GG | GG | CC | 0.000 272 | 0.0133 9 | **0.98633 8** | M | **BROW N** | **BROWN** |
| 123 | TT | GG | CC | GG | GG | CC | 0.000 272 | 0.0133 9 | **0.98633 8** | M | **BROW N** | **BROWN** |
| 124 | TT | GG | CA | GG | GG | CC | 0.000 272 | 0.0133 9 | **0.98633 8** | M | **BROW N** | **BROWN** |
| 125 | TT | GG | CC | GG | GG | CC | 0.000 272 | 0.0133 9 | **0.98633 8** | M | **BROW N** | **BROWN** |
| 126 | TT | GG | CC | GG | GG | CC | 0.000 | 0.0133 | **0.98633** | F | **BROW** | **BROWN** |

| | | | | | | | | | | | | |
|---|---|---|---|---|---|---|---|---|---|---|---|---|
| | | | | | | | 272 | 9 | **8** | | **N** | |
| 127 | TT | GG | CC | GG | GG | CC | 0.000272 | 0.01339 | **0.986338** | M | **BROWN** | **BROWN** |
| 128 | TT | GG | CC | GG | GG | CC | 0.000272 | 0.01339 | **0.986338** | M | **BROWN** | **BROWN** |
| 129 | CC | GG | CC | GG | GG | CC | **0.84781** | 0.087663 | 0.064527 | F | **BLUE** | **BLUE** |
| 130 | TT | GG | CC | GG | GG | CC | 0.000272 | 0.01339 | 0.986338 | M | **BROWN** | **BROWN** |
| 131 | CT | GG | CC | GG | GG | CC | 0.050323 | 0.113551 | 0.836126 | M | **BROWN** | **BROWN** |
| 132 | TT | GG | CC | GG | GG | CC | 0.000272 | 0.01339 | 0.986338 | M | **BROWN** | **BROWN** |
| 133 | CT | GG | CC | GG | GG | CC | 0.050323 | 0.113551 | 0.836126 | M | **BROWN** | **BROWN** |
| 134 | CC | GG | CC | GG | GG | CC | **0.84781** | 0.087663 | 0.064527 | M | **BLUE** | **BLUE** |
| 135 | CC | GG | CA | CC | GG | CC | 0.337744 | 0.114804 | 0.547452 | M | **BROWN** | **INTER** |
| 136 | CT | GG | CC | GG | GG | CC | 0.050323 | 0.113551 | 0.836126 | M | **BROWN** | **Brown** |
| 137 | CC | GG | CC | GG | GG | CC | **0.84781** | 0.087663 | 0.064527 | M | **BLUE** | **BLUE** |
| 138 | CC | GG | CA | CC | GG | CC | 0.337744 | 0.114804 | 0.547452 | M | **BROWN** | **INTER** |
| 139 | TT | GG | CC | GG | GG | CC | 0.000272 | 0.01339 | 0.986338 | M | **BROWN** | **BROWN** |
| 140 | TT | GG | CC | GG | GG | CC | 0.000272 | 0.01339 | 0.986338 | M | **BROWN** | **BROWN** |
| 141 | CT | GG | CC | GG | GG | CC | 0.050323 | 0.113551 | 0.836126 | M | **BROWN** | **BROWN** |
| 142 | CT | GG | AA | GC | AA | CC | 0.01223 | 0.050034 | 0.937736 | M | **BROWN** | **BROWN** |
| 143 | TT | GG | CC | GG | GG | CC | 0.000272 | 0.01339 | 0.986338 | F | **BROWN** | **BROWN** |
| 144 | CT | GG | AA | GC | AA | CC | 0.01223 | 0.050034 | 0.937736 | F | **BROWN** | **BROWN** |
| Swat | TT | GG | CC | GG | GG | CC | 0.000 | 0.0133 | 0.98633 | F | **BROW** | **BROWN** |

| | | | | | | | | | | | | |
|---|---|---|---|---|---|---|---|---|---|---|---|---|
| 145 | | | | | | | 272 | 9 | 8 | | **N** | |
| 146 | TT | GG | CC | GG | GG | CC | 0.000 272 | 0.0133 9 | 0.98633 8 | M | **BROW N** | **BROWN** |
| 147 | CT | GG | CC | GG | GG | CC | 0.050 323 | 0.1135 51 | 0.83612 6 | M | **BROW N** | **Brown** |
| 148 | TT | GG | CC | GG | GG | CC | 0.000 272 | 0.0133 9 | 0.98633 8 | F | **BROW N** | **BROWN** |
| 149 | CC | GG | CC | GG | GG | CC | **0.847 81** | 0.0876 63 | 0.06452 7 | M | **BLUE** | **BLUE** |
| 150 | CC | GG | CC | GG | GG | CC | **0.847 81** | 0.0876 63 | 0.06452 7 | M | **BLUE** | **BLUE** |

Table S 3: FROG- kb 6 SNP genotypes and colour prediction probabilities together with samples details of Pakhtoon population. Blue colour indicated Blue eye colour, green indicated Intermediate and brown colour indicated brown eye colour

| Sample ID | rs1800407 | rs168918 | rs1393350 | rs12913832 | rs12896399 | rs12203592 | P-Blue | P-Intermediate | P-Brown | Predicted eye | Actual Eye Colour |
|---|---|---|---|---|---|---|---|---|---|---|---|
| 1 | CT | G | CC | GC | GG | CC | 0.02417 | 0.08345 | 0.8924 | Brown | Brown |
| 2 | TT | A | AA | GC | GG | CC | 0.001808 | 0.03577 | 0.9624 | Brown | Brown |
| 3 | CT | GG | AA | GC | AA | CC | 0.9189 | 0.04365 | 0.03743 | Blue | Brown |
| 4 | TT | GG | CA | CC | GA | CC | 1.17E-04 | 0.007928 | 0.992 | Brown | Brown |
| 5 | CC | GG | CC | GG | GG | CC | 0.8702 | 0.07638 | 0.05347 | Blue | Blue |
| 6 | TT | GA | CA | CC | GG | CC | 2.37E-04 | 0.01735 | 0.9824 | Brown | Brown |
| 7 | CC | GG | CA | GC | GG | CC | 0.7799 | 0.08983 | 0.1303 | Blue | Blue |
| 8 | CC | GG | CC | GC | GG | CC | 0.6776 | 0.131 | 0.1915 | Blue | INTER |
| 9 | CC | GG | CC | GC | GG | CC | 0.6776 | 0.131 | 0.1915 | Blue | INTER |
| 10 | TT | GG | CA | GG | GG | CC | 2.37E-04 | 0.01735 | 0.9824 | Brown | Brown |
| 11 | CC | GG | AA | CC | GA | CC | 0.7055 | 0.08871 | 0.2058 | Blue | INTER |
| 12 | CC | GG | CA | CC | GG | CC | 0.4946 | 0.1254 | 0.38 | Blue | INTER |
| 13 | CC | GG | CA | CC | GG | CC | 0.367 | 0.1562 | 0.4768 | Brown | INTER |
| 14 | TT | GG | CC | GG | GG | CC | 2.37E-04 | 0.01735 | 0.9824 | Brown | Brown |
| 15 | CC | GG | CA | CC | GG | CC | 0.4946 | 0.1254 | 0.38 | Blue | INTER |
| 16 | CC | GG | AA | GC | GG | CC | 0.8566 | 0.0588 | 0.08464 | Blue | INTER |
| 17 | CC | GG | CC | GC | GG | CC | 0.6776 | 0.131 | 0.1915 | Blue | INTER |
| 18 | TT | GG | CA | GG | GG | CC | 7.17E-04 | 0.00613 | 0.9938 | Brown | Brown |
| 19 | CC | GG | CC | GG | GG | CC | 0.8702 | 0.07638 | 0.053447 | Blue | Blue |
| 20 | CC | GG | CC | GC | GG | CC | 0.6776 | 0.131 | 0.1915 | Blue | INTER |
| 21 | TT | GG | CC | GG | GG | CC | 7.17E-04 | 0.00613 | 0.9938 | Brown | BROWN |

| | | | | | | | | | | |
|---|---|---|---|---|---|---|---|---|---|---|
| 22 | CT | GG | CA | GG | GG | CC | 0.1496 | 0.1398 | 0.7105 | Brown | BROWN |
| 23 | CT | GG | CA | GC | GG | CC | 0.04017 | 0.08266 | 0.8772 | Brown | BROWN |
| 24 | TT | GA | CC | GG | GG | CC | 0.0027 | 0.0708 | 0.9264 | Brown | BROWN |
| 25 | TT | GG | CA | GC | GG | CC | 3.46E-04 | 0.01271 | 0.9869 | Brown | BROWN |
| 26 | TT | GG | CA | GG | GG | CC | 2.75E-05 | 0.02597 | 0.9731 | Brown | BROWN |
| 27 | CC | GG | CC | GG | GG | CC | 0.367 | 0.1562 | 0.4768 | Brown | BLUE |
| 28 | CT | GG | CC | GC | GA | CC | 0.03626 | 0.1041 | 0.8596 | Brown | BROWN |
| 29 | TT | GG | CC | GG | GA | CC | 0.001433 | 0.03338 | 0.9652 | Brown | BROWN |
| 30 | TT | GG | CC | GG | GG | CC | 0.001433 | 0.03338 | 0.9652 | Brown | BROWN |
| 31 | CT | GG | CC | GG | GG | CC | 4.48E-05 | 0.006084 | 0.9939 | Brown | BROWN |
| 32 | CT | GG | CC | GG | GG | CC | 0.005606 | 0.04262 | 0.9518 | Brown | BROWN |
| 33 | CT | GG | CA | GC | GG | CC | 0.02417 | 0.08345 | 0.8924 | Brown | BROWN |
| 34 | CT | GG | CA | GG | GG | CC | 0.1496 | 0.1398 | 0.7105 | Brown | BROWN |
| 35 | CT | GG | CC | GG | GG | CC | 0.09436 | 0.1479 | 0.7577 | Brown | BROWN |
| 36 | CT | GG | CC | GG | GG | CC | 0.09436 | 0.1479 | 0.7577 | Brown | INTER |
| 37 | CT | GG | CC | GG | GG | CC | 0.09436 | 0.1479 | 0.7577 | Brown | BROWN |
| 38 | CT | GG | CC | GC | GG | CC | 0.09436 | 0.1479 | 0.7577 | Brown | BROWN |
| 39 | CT | GG | CA | GG | GG | CC | 0.00944 | 0.04277 | 0.9478 | Brown | BROWN |
| 40 | CT | GG | CC | GC | GG | CC | 0.09436 | 0.1479 | 0.7577 | Brown | INTER |
| 41 | TT | GA | CC | GG | GA | CC | 0.004263 | 0.08972 | 0.906 | Brown | BROWN |
| 42 | CT | GG | CC | GG | GG | CC | 0.005606 | 0.04262 | 0.9518 | Brown | BROWN |
| 43 | TT | GG | CA | GG | GG | CC | 0.001567 | 0.02615 | 0.9723 | Brown | BROWN |
| 44 | TT | GG | CC | GG | GG | CC | 9.28E-04 | 0.02597 | 0.9731 | Brown | BROWN |
| 45 | CT | GG | AA | GG | GG | CC | 0.2291 | 0.1276 | 0.6433 | Brown | INTER |
| 46 | CC | GG | CC | GG | GG | CC | 0.8702 | 0.07638 | 0.05347 | Blue | BLUE |
| 47 | CT | GA | CC | GC | GG | CC | 0.063 | 0.1978 | 0.738 | Brow | BRO |

| | | | | | | | | | | | |
|---|---|---|---|---|---|---|---|---|---|---|---|
| | | | | | | | 42 | | 8 | n | WN |
| 48 | CT | GG | CC | GC | GA | CC | 0.03626 | 0.1041 | 0.8596 | Brown | BROWN |
| 49 | CT | GG | CC | GC | GG | CC | 0.02417 | 0.08345 | 0.8924 | Brown | INTER |
| 50 | TT | GG | CA | GC | GA | CC | 5.37E-04 | 0.01641 | 0.9831 | Brown | BROWN |
| 51 | CT | GG | CA | GG | GG | CC | 0.1496 | 0.1398 | 0.7105 | Brown | BROWN |
| 52 | CT | GA | CC | GC | GG | CC | 0.06342 | 0.1978 | 0.7388 | Brown | INTER |
| 53 | TT | GG | CC | GG | GG | CC | 9.28E-04 | 0.02597 | 0.9731 | Brown | BROWN |
| 54 | CT | GA | CC | GC | GA | CC | 0.09031 | 0.2343 | 0.6754 | Brown | INTER |
| 55 | TT | GG | CA | GG | GG | CC | 0.001567 | 0.02615 | 0.9723 | Brown | BROWN |
| 56 | CT | GG | CA | GG | GG | CC | 0.001567 | 0.02615 | 0.9723 | Brown | INTER |
| 57 | CT | GA | CA | GG | GG | CC | 0.2992 | 0.2526 | 0.4482 | Brown | INTER |
| 58 | CT | GG | CA | GG | GG | CC | 0.745 | 0.1198 | 0.1352 | Blue | BROWN |
| 59 | TT | GG | CC | GG | GG | CC | 9.28E-04 | 0.02597 | 0.9731 | Brown | BROWN |
| 60 | CT | GG | CC | GG | GG | CC | 0.09436 | 0.1479 | 0.7577 | Brown | INTER |
| 61 | CT | GG | CA | GC | GG | CC | 0.09436 | 0.1479 | 0.7577 | Brown | INTER |
| 62 | TT | GA | CC | GG | GG | CC | 0.002799 | 0.0708 | 0.9264 | Brown | BROWN |
| 63 | CT | GG | CA | GC | GG | CT | 0.04017 | 0.08266 | 0.8772 | Brown | BROWN |
| 64 | CT | GG | CC | CC | GG | CC | 0.005606 | 0.04262 | 0.9518 | Brown | BROWN |
| 65 | CT | GA | CA | CC | GG | CC | 0.0272 | 0.1113 | 0.8615 | Brown | BROWN |
| 66 | CT | GA | CC | CC | GG | CC | 0.01628 | 0.1118 | 0.8719 | Brown | BROWN |
| 67 | TT | GA | CC | CC | GG | CC | 1.40E-04 | 0.01722 | 0.9826 | Brown | BROWN |
| 68 | CT | GG | CC | GG | GG | CC | 0.09436 | 0.1479 | 0.7577 | Brown | BROWN |
| 69 | TT | GA | CA | GC | GG | CC | 0.00107 | 0.03553 | 0.9634 | Brown | BROWN |
| 70 | CC | GG | CC | GG | GG | CC | 0.6776 | 0.131 | 0.1915 | Blue | BLUE |
| 71 | TT | GA | CA | GG | GA | CC | 0.007182 | 0.09008 | 0.9027 | Brown | BROWN |
| 72 | TT | GG | CC | GG | GG | CC | 9.28E-04 | 0.02597 | 0.9731 | Brown | BROWN |

| | | | | | | | | | | | |
|---|---|---|---|---|---|---|---|---|---|---|---|
| 73 | CT | GG | CC | GG | GG | CC | 9436 | 0.1479 | 0.7577 | Brown | BROWN |
| 74 | TT | GG | CC | GC | GG | CC | 2.05E-04 | 0.01262 | 0.9872 | Brown | BROWN |
| 75 | CT | GG | CA | CC | GG | CC | 0.00944 | 0.04277 | 0.9478 | Brown | BROWN |
| 76 | CT | GG | CC | GC | GG | CC | 0.02417 | 0.08345 | 0.8924 | Brown | BROWN |
| 77 | TT | GG | CC | GG | GG | CC | 9.28E-04 | 0.02597 | 0.9731 | Brown | BROWN |
| 78 | CT | GG | CC | GG | GG | CC | 0.09436 | 0.1479 | 0.7577 | Brown | BROWN |
| 79 | CT | GG | CC | GG | GG | CC | 0.09436 | 0.1479 | 0.7577 | Brown | INTER |
| 80 | CT | GG | CC | CC | GG | CC | 0.005606 | 0.04262 | 0.9518 | Brown | BROWN |
| 81 | TT | GG | CC | GG | GG | CC | 9.28E-04 | 0.02597 | 0.9731 | Brown | BROWN |
| 82 | TT | GG | CC | GG | GG | CC | 9.28E-04 | 0.02597 | 0.9731 | Brown | BROWN |
| 83 | CT | GG | CC | GG | GG | CC | 0.09436 | 0.1479 | 0.7577 | Brown | BROWN |
| 84 | CC | GG | CC | GC | GG | CC | 0.6776 | 0.131 | 0.1915 | Blue | INTER |
| 85 | CT | GG | CC | GG | GG | CC | 0.09436 | 0.1479 | 0.7577 | Brown | BROWN |
| 86 | CT | GG | AA | GC | AA | CC | 0.1394 | 0.1183 | 0.7422 | Brown | BROWN |
| 87 | TT | GG | CC | GG | GG | CC | 9.28E-04 | 0.02597 | 0.9731 | Brown | BROWN |
| 88 | CT | GG | AA | GC | AA | CC | 0.1394 | 0.1183 | 0.7422 | Brown | BROWN |
| 89 | CC | GG | CC | GG | GG | CC | 0.8702 | 0.07638 | 0.05347 | Blue | BLUE |
| 90 | CT | GG | AA | GC | AA | CC | 0.1394 | 0.1183 | 0.7422 | Brown | BROWN |
| 91 | CT | GG | CC | GG | GG | CC | 0.09436 | 0.1479 | 0.7577 | Brown | BROWN |
| 92 | CT | GG | CC | GG | GG | CC | 0.09436 | 0.1479 | 0.7577 | Brown | INTER |
| 93 | TT | GG | CC | GG | GG | CC | 9.28E-04 | 0.02597 | 0.9731 | Brown | BROWN |
| 94 | TT | GG | CA | GG | GG | CC | 0.001567 | 0.02615 | 0.9723 | Brown | BROWN |
| 95 | CC | GG | CC | GG | GG | CC | 0.8702 | 0.07638 | 0.05347 | Blue | BLUE |
| 96 | CT | GG | CC | GG | GG | CC | 0.09436 | 0.1479 | 0.7577 | Brown | BROWN |
| 97 | TT | GG | CA | GG | GG | CC | 0.001567 | 0.02615 | 0.9723 | Brown | BROWN |
| 98 | TT | GG | CC | GG | GG | CC | 9.28E | 0.02597 | 0.973 | Brow | BRO |

| | | | | | | | | | | | |
|---|---|---|---|---|---|---|---|---|---|---|---|
| | | | | | | | -04 | | 1 | n | WN |
| 99 | TT | GG | CA | GG | GG | CC | 0.001567 | 0.02615 | 0.9723 | Brown | BROWN |
| 100 | CC | GG | CA | CC | GG | CC | 0.4946 | 0.1254 | 0.38 | Blue | INTER |
| 101 | CT | GG | CC | GG | GG | CC | 0.09436 | 0.1479 | 0.7577 | Brown | BROWN |
| 102 | CT | GG | AA | GC | AA | CC | 0.1394 | 0.1183 | 0.7422 | Brown | BROWN |
| 103 | TT | GG | CC | GG | GG | CC | 9.28E-04 | 0.02597 | 0.9731 | Brown | BROWN |
| 104 | TT | GG | CA | GG | GG | CC | 0.001567 | 0.02615 | 0.9723 | Brown | BROWN |
| 105 | CT | GG | CC | GG | GG | CC | 0.09436 | 0.1479 | 0.7577 | Brown | BROWN |
| 106 | TT | GG | CC | GG | GG | CC | 9.28E-04 | 0.02597 | 0.9731 | Brown | BROWN |
| 107 | CT | GG | AA | GC | AA | CC | 0.1394 | 0.1183 | 0.7422 | Brown | BROWN |
| 108 | CT | GG | CC | GG | GG | CC | 0.09436 | 0.1479 | 0.7577 | Brown | BROWN |
| 109 | TT | GG | CC | GG | GG | CC | 9.28E-04 | 0.02597 | 0.9731 | Brown | BROWN |
| 110 | TT | GG | CC | GG | GG | CC | 0.09436 | 0.1479 | 0.7577 | Brown | BROWN |
| 111 | CT | GG | AA | GC | AA | CC | 0.1394 | 0.1183 | 0.7422 | Brown | BROWN |
| 112 | TT | GG | CC | GG | GG | CC | 9.28E-04 | 0.02597 | 0.9731 | Brown | BROWN |
| 113 | CT | GG | CC | GG | GG | CC | 0.09436 | 0.1479 | 0.7577 | Brown | BROWN |
| 114 | TT | GG | CC | GG | GG | CC | 0.09436 | 0.1479 | 0.7577 | Brown | BROWN |
| 115 | TT | GG | CC | GG | GG | CC | 0.09436 | 0.1479 | 0.7577 | Brown | BROWN |
| 116 | CT | GG | CC | GG | GG | CC | 0.09436 | 0.1479 | 0.7577 | Brown | BROWN |
| 117 | TT | GG | CC | GG | GG | CC | 9.28E-04 | 0.02597 | 0.9731 | Brown | BROWN |
| 118 | CT | GG | AA | GC | AA | CC | 0.1394 | 0.1183 | 0.7422 | Brown | BROWN |
| 119 | CC | GG | CA | CC | GG | CC | 0.4946 | 0.1254 | 0.38 | Blue | INTER |
| 120 | CT | GG | CC | GG | GG | CC | 0.09436 | 0.1479 | 0.7577 | Brown | BROWN |
| 121 | TT | GG | CC | GG | GG | CC | 9.28E-04 | 0.02597 | 0.9731 | Brown | BRWON |
| 122 | TT | GG | CA | GG | GG | CC | 0.001567 | 0.02615 | 0.9723 | Brown | BROWN |
| 123 | TT | GG | CC | GG | GG | CC | 9.28E-04 | 0.02597 | 0.9731 | Brown | BROWN |

| | | | | | | | | | | | |
|---|---|---|---|---|---|---|---|---|---|---|---|
| 124 | TT | GG | CA | GG | GG | CC | 0.001567 | 0.02615 | 0.9723 | Brown | BROWN |
| 125 | TT | GG | CC | GG | GG | CC | 9.28E-04 | 0.02597 | 0.9731 | Brown | BROWN |
| 126 | TT | GG | CC | GG | GG | CC | 9.28E-04 | 0.02597 | 0.9731 | Brown | BROWN |
| 127 | TT | GG | CC | GG | GG | CC | 9.28E-04 | 0.02597 | 0.9731 | Brown | BROWN |
| 128 | TT | GG | CC | GG | GG | CC | 9.28E-04 | 0.02597 | 0.9731 | Brown | BROWN |
| 129 | CC | GG | CC | GG | GG | CC | 0.8702 | 0.07638 | 0.05347 | Blue | BLUE |
| 130 | TT | GG | CC | GG | GG | CC | 9.28E-04 | 0.02597 | 0.9731 | Brown | BROWN |
| 131 | CT | GG | CC | GG | GG | CC | 0.09436 | 0.1479 | 0.7577 | Brown | BROWN |
| 132 | TT | GG | CC | GG | GG | CC | 9.28E-04 | 0.02597 | 0.9731 | Brown | BROWN |
| 133 | CT | GG | CC | GG | GG | CC | 0.09436 | 0.1479 | 0.7577 | Brown | BROWN |
| 134 | CC | GG | CC | GG | GG | CC | 0.8702 | 0.07638 | 0.05347 | Blue | BLUE |
| 135 | CC | GG | CA | CC | GG | CC | 0.4946 | 0.1254 | 0.38 | Blue | INTER |
| 136 | CT | GG | CC | GG | GG | CC | 0.09436 | 0.1479 | 0.7577 | Brown | Brown |
| 137 | CC | GG | CC | GG | GG | CC | 0.8702 | 0.07638 | 0.05347 | Blue | BLUE |
| 138 | CC | GG | CA | CC | GG | CC | 0.4946 | 0.1254 | 0.38 | Blue | INTER |
| 139 | TT | GG | CC | GG | GG | CC | 9.28E-04 | 0.02597 | 0.9731 | Brown | BROWN |
| 140 | TT | GG | CC | GG | GG | CC | 9.28E-04 | 0.02597 | 0.9731 | Brown | BROWN |
| 141 | CT | GG | CC | GG | GG | CC | 0.09436 | 0.1479 | 0.7577 | Brown | BROWN |
| 142 | CT | GG | AA | GC | AA | CC | 0.001808 | 0.03577 | 0.9624 | Brown | BROWN |
| 143 | TT | GG | CC | GG | GG | CC | 9.28E-04 | 0.02597 | 0.9731 | Brown | BROWN |
| 144 | CT | GG | AA | GC | AA | CC | 0.001808 | 0.03577 | 0.9624 | Brown | BROWN |
| 145 | TT | GG | CC | GG | GG | CC | 9.28E-04 | 0.02597 | 0.9731 | Brown | BROWN |
| 146 | TT | GG | CC | GG | GG | CC | 9.28E-04 | 0.02597 | 0.9731 | Brown | BROWN |
| 147 | CT | GG | CC | GG | GG | CC | 0.09436 | 0.1479 | 0.7577 | Brown | BROWN |
| 148 | TT | GG | CC | GG | GG | CC | 9.28E-04 | 0.02597 | 0.9731 | Brown | BROWN |
| 149 | CC | GG | CC | GG | GG | CC | 0.870 | 0.07638 | 0.053 | Blue | BLUE |

| | | | | | | | | | | | |
|---|---|---|---|---|---|---|---|---|---|---|---|
| | | | | | | | 2 | | 47 | | |
| 150 | CC | GG | CC | GG | GG | CC | 0.870<br>2 | 0.07638 | 0.053<br>47 | Blue | BLUE |
